# Risk of serious infections in multiple sclerosis patients by disease course and disability status: Results from a Swedish register-based study

**DOI:** 10.1016/j.bbih.2022.100470

**Published:** 2022-05-11

**Authors:** Judith S. Brand, Kelsi A. Smith, Fredrik Piehl, Tomas Olsson, Scott Montgomery

**Affiliations:** a Clinical Epidemiology and Biostatistics, School of Medical Sciences, Örebro University, Sweden; b Clinical Epidemiology Division, Karolinska Institutet, Stockholm, Sweden; c Department of Clinical Neuroscience, Karolinska Institutet, Stockholm, Sweden; d Department of Epidemiology and Public Health, University College London, UK

**Keywords:** Multiple sclerosis, Serious infections, Disease course and disability, National register-based study

## Abstract

**Background and objectives:**

Serious infections are an emerging concern with increasing use of potent immunomodulation in multiple sclerosis (MS), but the extent to which MS disease features influence infectious susceptibility is poorly characterized. The objective of this study was to assess the associations of MS disease course and disability status with risk of serious infections.

**Methods:**

A cohort of 8660 MS patients was individually matched on age, sex and region of residence with 86,600 people without MS from the general population using national registers in Sweden. The study period was from 1996 to 2012, with follow-up until December 31, 2014. The main outcomes were infection as the underlying or contributory cause of death or infection-related hospital admission identified in the Cause of Death and Patient registers. MS disease course (relapsing-remitting or progressive disease) and Expanded Disability Status Scale (EDSS) score (six and over or below six) were extracted from the MS Register Hazard ratios (HRs) for any serious infection were estimated using flexible parametric models.

**Results:**

During a median follow-up of 9.6 years (interquartile range = 5.5–13.5 years), 1337 MS patients experienced a serious infection. Compared with individually matched people without MS, risk of serious infection was greater for progressive disease (HR = 3.80; 95% CI 3.52: 4.09) than relapsing-remitting disease (HR = 1.77; 95% CI: 1.62:1.93). A similar pattern of risk was seen for dichotomised EDSS score (HR = 4.26; 95% CI 3.87: 4.70 for EDSS 6.0–9.5 and HR = 1.30; 95% CI 1.1853: 1.43 for EDSS 0.0–5.5). Overall, associations with greater disability did not notably differ by immunomodulatory therapy use, but associations with lower disability were more pronounced in patients receiving these therapies.

**Conclusions:**

Disease course or EDSS score (which may be more readily available than MS course in some patients) should be considered in individual management and monitoring of MS patients, including assessing benefit-risk of therapies that influence general immune function.

## 1 Introduction

Multiple sclerosis (MS) patients are at increased risk of serious infections, reflected by infection-related hospital admissions and cause-specific mortality (Marrie et al., 2014; Montgomery et al., 2013; Smestad et al., 2009; Burkill et al., 2017). Clinically manifest infections can have a significant influence on the lives of MS patients as they have been associated with relapses resulting in more sustained neurological deficits (Buljevac et al., 2002). Serious infections have become a greater concern recently due to the introduction of newer disease modifying therapies (DMTs), which not only improve disease control, but also increase the susceptibility to infections (Br ü ck et al., 2013; Luna et al., 2020). Limited evidence, however, exists regarding other MS-specific predictors of infection risk among patients. The contribution of non-treatment specific factors is indirectly supported by data showing an increased infection rate both immediately before and after MS diagnosis (Castelo-Branco et al., 2020). Whilst infections may occur at any time point during the course of the disease, it is anticipated that the susceptibility to infections increases with longer disease duration and more advanced disability. Indeed, MS-related functional loss including bladder dysfunction and respiratory problems have been identified as risks for infection (Celius, 2017). Despite this variation in infection susceptibility, to the best of our knowledge, no population-based data exist for the risk of serious infections by disease course (relapsing-remitting vs. progressive) or Expanded Disability Status Scale (EDSS) (Kurtzke, 1983) score, the most widely used MS outcome measures in clinical practice. Because such information is pertinent for guiding individual patient management and monitoring, including among patients receiving DMTs, we assessed the association of these clinically available measures with the risk of serious infections in a well-characterised Swedish cohort of MS patients and matched reference individuals from the general population.

## 2 Methods

This is a matched cohort study of patients in the Swedish Multiple Sclerosis Register (MSR), covering approximately 80% of MS patients diagnosed in Sweden (Hillert and Stawiarz, 2015), with a high specificity for an MS diagnosis based on cerebrospinal fluid oligoclonal band data (Bahmanyar et al., 2009; Alping et al., 2019). Each patient was matched with 10 individuals without MS based on sex, year of birth, vital status and region of residence at the time of MS diagnosis. Some 8660 patients with a first MS diagnosis between 1996 and 2012 with the date retrieved from the MSR or Patient Register, with information on clinical course (relapsing-remitting or progressive, with year of transition) in the MSR were included, and their corresponding matched individuals without MS (n = 86,600). Progressive disease was defined as either a primary or secondary progressive course and for patients with secondary progressive MS the year of transition was extracted if available. Patients diagnosed with progressive-relapsing disease were not part of our study population, as their number was too small for meaningful analysis, and this diagnosis is no longer recorded as a separate disease course category in the MSR. Individual linkage with the Patient Register (Ludvigsson et al., 2011), the Cause of Death Register (Brooke et al., 2017) and the Total Population Register (Ludvigsson et al., 2016) was performed using the unique personal identification number issued to all residents in Sweden with follow-up to December 31, 2014.

Ethical approval for this study was given by the Regional Ethical Review Board at Karolinska Institutet (2013/1156-31/5). Informed consent is required for inclusion in the MSR.

### 2.1 Outcomes

The main outcome was any serious infection after cohort entry, defined as a diagnosis of infection associated with hospital admission (including primary and secondary discharge diagnoses) as recorded in the Patient Register (with national coverage of diagnoses included in this study (Ludvigsson et al., 2011)) or mortality due to infection (as underlying or contributory cause) as recorded in the Cause of Death Register. As a secondary outcome, we studied serious infections subdivided by site into 6 groups: respiratory tract infections, sepsis, infections of the central nervous system, skin infections, gastrointestinal infections, and urinary tract infections, identified using the international classification of diseases, 9th and 10th revision (Supplementary Table 1).

### 2.2 MS disease course and EDSS score

The MSR holds information on disease course at diagnosis (defined as either relapse-remitting or primary progressive disease) as well as the recorded year of transition to secondary progressive disease. The EDSS is also recorded prospectively as assessed by the neurologist at routine clinical care visits, and ranges from 0 to 9.5 with 0.5 unit increments with higher scores indicating greater disability (Kurtzke, 1983). EDSS scores were categorised as either mild/moderate disability (0.0–5.5) or severe disability (6.0–9.5).

### 2.3 Covariates

We retrieved the following covariates from the registry data: age, sex, calendar year of cohort entry, region of residence, educational attainment (compulsory school or less, upper secondary education, higher education, unknown) and for MS patients also information on DMT use. Only DMTs known to have a general impact on the immune system (Rituximab, Natalizumab, Alemtuzumab, Dimethyl Fumarate or Fingolimod) were extracted from the MSR and categorised as ever or never used.

### 2.4 Statistical analyses

All individuals were followed from the date of cohort entry (date of diagnosis for MS patients and equivalent time point in the matched comparators) until the outcome (infection-related hospital admission or mortality), death by other causes, emigration, or end of the study period (December 31, 2014), whichever occurred first. Flexible parametric models (Royston and Parmar, 2002) were used to allow the association between exposure and outcome to vary over time. In all models, attained age (i.e. the actual age of the individual at follow-up) was the underlying time scale and a restricted cubic spline with five degrees of freedom was used for the baseline hazard. Time-dependent effects were modelled by adding an interaction term with time using a second spline with three degrees of freedom. To facilitate comparisons across the different models, HRs are also presented for predefined strata of attained age (<40 years, 40–60 years, > 60 years). All analyses were adjusted for matching factors (age, sex and region of residence) and additionally for educational attainment and calendar period of cohort entry. (1996–1999, 2000–2003, 2004–2007, 2008–2012). Disease course and EDSS scores were modelled as time-varying variables to account for transitions in disease status during follow-up. To avoid problems associated with multicollinearity, disease course and EDSS scores were not included in the same model and only assessed separately. We also examined associations with infection-related deaths and hospital admissions separately. To assess the extent to which results differed by DMTs we repeated all analyses by DMT treatment (ever vs. never).

We also stratified analyses by sex as previous studies have demonstrated the risk of serious infections to be higher among male than female MS patients (Montgomery et al., 2013; Wijnands et al., 2017; Persson et al., 2020). To address possible surveillance bias, we conducted a sensitivity analysis restricted to serious infections with a main diagnosis only. Since primary diagnoses of infection will predominantly capture community-acquired infections, this analysis also provides some insight into the extent to which associations are driven by community acquired infections rather than infections diagnosed incidentally in hospital. We further evaluated associations with more severe recurrent infections, defined as at least 2 diagnoses of infection-related hospital admission more than 6 months apart. All data were analyzed using Stata version 15 MP (StataCorp, College Station, TX, USA).

## 3 Results

The median age at MS diagnosis was 39 years, and 70.6% of patients were female. Patients with MS had somewhat higher educational attainment than the cohort without MS (Table 1).

**Table 1:** Characteristics of the study population (N = 95260).

	MS (n = 8660)	No MS (n = 86600)
Sex, % (N)		
Male	29.4 (2548)	29.4 (25480)
Female	70.6 (6112)	70.6 (61120)
Calendar year at cohort entry, % (N)		
1996–1999	17.3 (1494)	17.3 (14940)
2000–2003	29.4 (2547)	29.4 (25470)
2004–2007	23.7 (2051)	23.7 (20510)
2008–2012	29.7 (2568)	29.7 (25680)
Age at cohort entry (years), % (N)		
< 18	1.6 (138)	1.5 (1325)
18-40	51.7 (4476)	51.9 (44926)
41-64	44.1 (3815)	43.9 (38035)
≥ 65	2.7 (231)	2.7 (2314)
Educational level, % (N)		
Compulsory school or less	13.2 (1141)	14.3 (12366)
Upper secondary	46.4 (4021)	45.7 (39545)
Higher education	39.9 (3457)	39.2 (33927)
Unknown	0.5 (41)	0.9 (762)
Disease course at the last clinical visit, % (N) *		
Relapsing-remitting	65.4 (5664)	–
Secondary progressive	25.0 (2169)	–
Primary progressive	9.5 (827)	–
Last recorded EDSS score, % (N) *		
0.0–2.5 (mild)	52.0 (3969)	
3.0–5.5 (moderate)	25.1 (1911)	–
6.0–9.5 (severe)	22.9 (1746)	–
Missing	11.9 (1034)	–

Abbreviations: MS = multiple sclerosis; EDSS = Expanded Disability Status Scale. Last recorded disease status (course/EDSS) during follow-up.

In total, 5664 MS patients (65.4%) had RRMS and did not develop progressive disease during follow-up. An EDSS score was recorded for 7589 patients (87.6%) and of these 1912 (25.2%) had greater disability (EDSS 6.0–9.5). Information on DMT use was available for 7382 (85.2%) patients.

Median follow-up was 9.6 years (Q1-Q3 = 5.5–13.5 years) and the median attained age at analysis was 49.6 years (Q1-Q3 = 40.3–59.5 years). During this period of follow-up, 1337 patients with MS had any serious infection, and a total of 1331 infection-related hospital admissions and 93 infection-related deaths were recorded. The rate of serious infection was 32.6 and 9.8 per 1000 person-years among patients with progressive and relapsing-remitting disease respectively compared to 6.75 per 1000 person-years in matched reference individuals without MS. This translates to an increased relative risk of serious infections among MS patients, particularly in patients with a progressive course (HR = 3.80; 95% CI 3.52: 4.09) but also in those with relapsing-remitting disease (HR = 1.77; 95% CI 1.62:1.93). A similar pattern of association was seen for EDSS score divided into below six and six or above (HR = 4.26; 95% CI 3.87: 4.70 for EDSS 6.0–9.5 and HR = 1.30; 95% CI 1.18: 1.43 for EDSS 0.0–5.5) compared with individuals without MS. Stratified analyses by sex revealed that relative risks associated with more advanced disease and disability were greater in male than female MS patients (Supplementary Table 2).

**Fig. 1:**
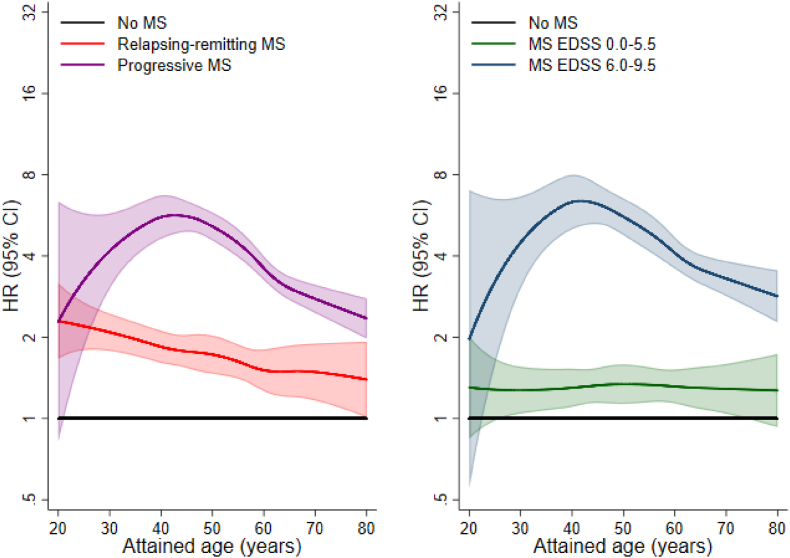
Relative risks of serious infection associated with MS disease course and disability by attained age. Hazard ratios comparing rates of serious infections in MS patient groups by disease course and disability with rates in matched individuals from the general population without MS (= reference). All hazard ratios are derived from flexible parametric models with attained age as underlying time scale and are adjusted for matching factors (age, sex and region of residence) and educational attainment and calendar period of cohort entry.AbbreviationsMS = multiple sclerosis; EDSS = Expanded Disability Status Scale; HR = hazard ratio; CI = confidence interval.

Analyses stratified by attained age are presented in Fig. 1 and Table 2. Overall, HRs for infection associated with progressive disease and greater disability declined with increasing attained age. Patients with progressive disease who were aged <40 years, had a 4.7-fold increased risk of serious infection (HR = 4.68; 95% CI 3.63: 6.03) compared to individuals without MS in the same age group. HRs for patients with progressive disease aged 40–60 years and >60 years were 4.21 (95% CI 3.78: 4.69) and 2.84 (95% CI 2.52: 3.19) respectively.

**Table 2:** Associations of MS disease course and disability with risk of any serious infection, overall and by attained age.

	Overall			Attained age					
				<40 yrs		40–60 yrs		>60 yrs	
	Total N	N events	HR (95% CI)	N events	HR (95% CI)	N events	HR (95% CI)	N events	HR (95% CI)
No MS	86600	5576	REF	1304	REF	2413	REF	1859	REF
MS clinical course									
Relapsing-remitting	5664	536	1.77 (1.62; 1.93)	234	2.03 (1.76; 2.33)	238	1.67 (1.46; 1.91)	64	1.56 (1.21; 2.00)
Progressive	2996	801	3.80 (3.52; 4.09)	64	4.68 (3.63; 6.03)	394	4.21 (3.78; 4.69)	343	2.84 (2.53; 3.19)
MS EDSS score									
0.0–5.5 (mild/moderate)	5677	440	1.30 (1.18; 1.43)	136	1.24 (1.04; 1.48)	206	1.31 (1.13; 1.51)	98	1.39 (1.14; 1.71)
6.0–9.5 (severe)	1912	449	4.26 (3.87; 4.70)	44	5.42 (4.00; 7.34)	212	5.17 (4.48; 5.95)	193	3.42 (2.95; 3.97)

Hazard ratios comparing rates of serious infections in MS patient groups by disease course and disability with rates in matched individuals from the general population without MS (= reference). All hazard ratios are derived from flexible parametric models and are adjusted for matching factors (age, sex and region of residence) and educational attainment and calendar period of cohort entry.

Abbreviations: MS = multiple sclerosis; EDSS = Expanded Disability Status Scale; HR = hazard ratio; CI = confidence interval; REF = reference category.

**Fig. 2:**
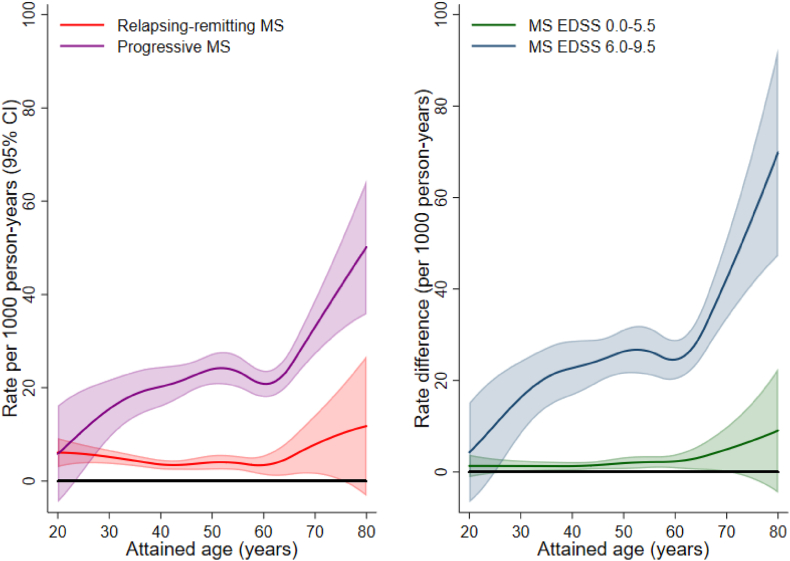
Absolute rate differences of serious infection associated with MS disease course and disability by attained age. Differences in absolute rates of serious infection associated with MS disease course and disability status. All rates are derived from flexible parametric models with attained age as underlying time scale using matched individuals from the general population without MS as a reference. Abbreviations: MS = multiple sclerosis; EDSS = Expanded Disability Status Scale; CI = confidence interval.

Absolute rates of serious infections for the patient groups and individuals without MS are plotted as a function of attained age in Supplementary Fig. 1 with corresponding rate differences in Fig. 2. Whereas HRs for progressive disease and greater disability decreased with increasing age, absolute rate differences increased exponentially with age (Fig. 2).

After the age of 60 years the rate of serious infection in patients with a progressive course and age-matched comparators was 41.7 (95 CI 37.5: 46.4) and 14.7 (95% CI 14.1: 15.4), respectively, per 1000 person-years. Corresponding rates before the age of 40 years were 21.7 (95% CI 17.0: 27.7) and 4.8 (95% CI 4.5: 5.0), respectively (Supplementary Table 3).

Analyses by infection site showed that relative risks and absolute rate differences for serious infections associated with MS disease severity were highest for urinary tract infections (Supplementary Table 4). Separate analyses for each infection type revealed that the overall results were mainly driven by infection-related hospital admissions. Analysis of infection-related mortality was limited by small numbers and showed elevated risks among patients with progressive disease and greater disability, but not among those with relapsing-remitting disease and lower disability scores (Supplementary Table 5).

Associations with progressive disease were not notably different among those who were treated with a DMT among the subset of patients where information on DMT use was available (HR = 4.37; 95% CI 3.52: 5.42) and those who were not (HR = 3.76; 95% CI 3.41: 4.14). Associations with relapsing-remitting disease, on the other hand, were of somewhat higher magnitude in those treated with a DMT (HR = 2.22; 95% CI 1.95: 2.53) than those who were not (HR = 1.49; 95% CI 1.31: 1.70). The pattern of association was similar for EDSS scores and in patients not receiving a DMT the association with lower scores was only evident at older attained age (Table 3).

**Table 3:** Associations of MS disease course and disability with risk of serious infection stratified by disease modifying therapy, overall and by attained age.

	Overall			Attained age					
				<40 yrs		40–60 yrs		>60 yrs	
	Total N	N events	HR (95% CI)	N events	HR (95% CI)	N events	HR (95% CI)	N events	HR (95% CI)
Never DMT									
MS clinical course									
Relapsing-remitting	2601	229	1.49 (1.31; 1.70)	62	1.39 (1.07; 1.79)	126	1.52 (1.27; 1.82)	41	1.64 (1.20; 2.24)
Progressive	1667	454	3.76 (3.41; 4.14)	39	5.94 (4.29; 8.23)	232	3.98 (3.47; 4.57)	183	2.82 (2.42; 3.29)
MS EDSS score									
0.0–5.5 (mild/moderate)	2706	197	1.10 (0.96; 1.27)	29	0.69 (0.48; 1.00)	108	1.16 (0.95; 1.40)	60	1.38 (1.07; 1.79)
6.0–9.5 (severe)	954	279	4.35 (3.85; 4.91)	28	7.52 (5.14; 11.00)	130	4.75 (3.98; 5.68)	121	3.72 (3.09; 4.47)

Ever DMT									
MS clinical course									
Relapsing-remitting	2592	250	2.22 (1.95; 2.53)	162	2.49 (2.11; 2.93)	83	1.91 (1.53; 2.38)	5	1.82 (0.75; 4.39)
Progressive	522	84	4.37 (3.52; 5.42)	20	3.81 (2.45; 5.94)	55	4.61 (3.52; 6.03)	9	3.49 (1.81; 6.74)
MS EDSS score									
0.0–5.5 (mild/moderate)	2396	182	1.63 (1.41; 1.90)	103	1.64 (1.34; 2.01)	73	1.62 (1.28; 2.05)	6	1.93 (0.86; 4.30)
6.0–9.5 (severe)	434	64	5.18 (4.05; 6.63)	15	3.86 (2.31; 6.43)	43	6.32 (4.67; 8.55)	6	3.54 (1.59; 7.92)

Hazard ratios comparing rates of serious infections in MS patient groups by disease course and disability and stratified by disease modifying therapy with rates in matched individuals from the general population without MS (= reference). All hazard ratios are derived from flexible parametric models and are adjusted for the matching factors (age sex and region of residence) and for educational attainment and calendar period of cohort entry.

Abbreviations: MS = multiple sclerosis; EDSS = Expanded Disability Status Scale; HR = hazard ratio; CI = confidence interval; REF = reference category, DMT, disease modifying therapy. DMT is defined as Rituximab, Natalizumab, Alemtuzumab, Dimethyl Fumarate or Fingolimod.

Restricting analyses to primary diagnoses of infections produced similar results to those observed in main analyses, overall and stratified by DMT treatment (Supplementary Tables 6 and 7). Also, results for the risk of infection-related hospitalisation were not notably different in analyses examining recurrent infections, although associations with progressive disease and greater disability were of higher magnitude (Supplementary Table 8).

## 4 Discussion

In this large nationwide cohort study, the raised risk of serious infections in MS patients, compared to people without MS, was most notable among those with progressive disease and greater disability as indicated by a higher EDSS score. This pattern of increased infection susceptibility with disease severity was observed in MS patients treated with and without DMTs and was more pronounced among male than female patients. In addition, we found that whilst the relative risk of serious infections compared with the general population was higher among younger MS patients, greater absolute rates were seen at older ages.

### 4.1 Comparison with existing literature

Whilst the hospital admission rate among MS patients has declined in recent years, infections remain a common cause of hospital admission in this patient population (Marrie et al., 2014) and this number may rise considering the increasing incidence of MS and life expectancy of people living with MS (Magyari and Sorensen, 2019). Whilst functional limitations are generally regarded as a key risk factor for infections, their association with the incidence of serious infections has rarely been explicitly examined in a population-based setting. A recent study (Pirttisalo et al., 2020) found that MS patients with a hospital admission for infections more often had progressive disease and advanced disability, but could not examine absolute risks or risk patterns by infection site and other susceptibility factors including DMTs. Here we show that relative and absolute excess risks associated with a progressive course and greater disability are greatest for urinary tract infections. Most people with MS experience bladder dysfunction during their lives including possible urinary retention and catheter use, and this functional limitation has been shown to correlate strongly with the level of disability (Lin et al., 2019; Nortvedt et al., 2007; de S è ze et al., 2007). Previous studies have also demonstrated a similar site-specific pattern of infections among MS patients in general with a particular high risk of urinary tract infections (Montgomery et al., 2013; Castelo-Branco et al., 2020; Wijnands et al., 2017). Our data further indicate that the raised risk of serious infections with greater functional loss is independent of DMT use. Clinically, this finding suggests that disability status can be used to identify patients who are already at risk of infection because of DMTs. Whilst associations with progressive disease and higher EDSS scores did not notably differ by DMT use, associations with relapsing remitting disease and lower EDSS scores were less pronounced in patients who did not receive a DMT. This apparent difference in association by DMT use is most likely due to differences in disease severity, with patients with milder scores or a relapsing remitting course having more severe disease when treated with a DMT.

Previous studies (Montgomery et al., 2013; Wijnands et al., 2017; Persson et al., 2020) have demonstrated disproportionally higher risks of serious infections in male than female MS patients, which has been attributed to a more rapid and aggressive disease course in male patients (Tremlett et al., 2006; Menon et al., 2013). Here we show that whilst higher magnitude associations are found among male patients, a raised risk of serious infections with progressive course and greater disability exists in both males and females, suggesting that a greater male susceptibility for infections in general (Klein and Flanagan, 2016) is a more likely explanation for this observation.

We further found lower magnitude HRs with increasing attained age. This pattern can be explained by the increasing baseline incidence of infections with advancing age, especially when age-related consequences occur more frequently among the general population. Similarly, serious infections are likely to be rarer among younger than older MS patients, explaining the greater absolute risk observed with older age. In light of recent trends in MS disease presentation with increasing age at onset and longer life expectancy (Magyari and Sorensen, 2019), more patients with MS will experience serious infections.

Overall, we found that risks of infection-related hospital admission and mortality followed a similar pattern with disease severity, although the rate of infection-related death was not increased among patients with relapsing-remitting disease. It should, however, be noted that the number of deaths due to infection was relatively small in our study and this reflects clinical reality: excess mortality in MS patients continues to exist, but the gap with the general population has narrowed (Burkill et al., 2017). However, it is possible that this trend may be interrupted by increasing use of newer therapeutic options such as alemtuzumab, ocrelizumab, ofatumumab and rituximab, which are associated with an increased risk of opportunistic life-threatening infections (Zappulo et al., 2019). In addition, the rare but serious infection progressive multifocal leukoencephalopathy (PML) occurs in people with immunosuppression, but MS patients were not at notably raised risk of PML until introduction of natalizumab (Iacobaeus et al., 2018). The lack of PML among MS patients in Sweden in recent years is testament to the value of planning treatment and monitoring based on screening of risk factors for infection (K å gstr ö m et al., 2021).

### 4.2 Strengths and limitations

A major strength of this study is the nationwide register-based design with prospectively recorded data, minimising potential selection and information biases. Another strength is the high coverage and specificity of MS diagnoses in the SMR with only a small proportion of patients having missing data on MS-related clinical characteristics. The use of flexible parametric models enabled us to model relative and absolute risks of serious infections across the entire attained age range. This reduces reliance on the exact date of MS onset and allows estimation of risk estimates at ages with the highest population burden of serious infections. The outcome of this study defined as infections associated with hospital admission or death is suited to study infections that are potentially life-threatening (as opposed to infections among outpatients) and clinically most relevant in this patient population. Our study also has potential limitations. Compared to the general population, MS patients may have more frequent healthcare contacts resulting in an infection diagnosis. Surveillance and referral bias could partly explain the excess risk of infection-related hospital admission among MS patients, but not associations with infection-related mortality. Also, this bias would be more plausible for less serious infections that otherwise would have gone unnoticed. Hence, results of our sensitivity analyses focusing on main infection diagnoses only and recurrent infection-related hospital admissions suggest that the influence of this possible bias is minimal. Another limitation is the possible misclassification of the outcome. An external review study of the Swedish Inpatient Register (Ludvigsson et al., 2011) found high coverage and validity for most hospital diagnoses, but infectious diseases have not been extensively validated. A Danish validation study (Henriksen et al., 2014), however, has shown that hospital discharge diagnoses are reliable for detecting infections, with a high degree of validity for site-specific infections and moderate validity for overall infections (sensitivity = 80% and specificity = 84%). In our study, associations were also not notably different in analyses limited to main diagnoses only (i.e. infection as primary discharge diagnosis or underlying cause of death), reducing the likelihood of potential bias due to misclassification. We did not have information on some relevant lifestyle factors, such as smoking status, and vaccination rates in MS patients and their matched comparators that could affect the associations observed. However, it is unlikely that such factors fully explain the greater risk of serious infections with disease progression and more advanced disability including the site-specific risk pattern observed. Lastly, MS treatment has changed considerably during the study period and several new drugs have been introduced in recent years: the list of DMTs we used was not exhaustive, and some treatments would have been rarely used during our study period, such as alemtuzumab and dimethyl fumarate. DMTs may also have a therapeutic lag before infectious complications become fully manifest. Hence, our results may not be directly applicable to other settings with long-term administration of more potent DMTs, since the proportion of patients treated with such treatments was only modest in this study. Despite these potential limitations, there was no notable evidence that DMT use explains the overall associations of MS disease course and disability with risk of serious infections.

### 4.3 Implications of the research

Our study underscores the notion that susceptibility to serious infections is determined by disease-specific markers that are readily available in clinical practice. This observation calls for more awareness among healthcare professionals and may have implications for management of patients with MS treated with or without DMTs. For instance, it has been suggested that prior infectious history should be taken in consideration when selecting a therapeutic strategy and potential precautionary measures in MS patients (Montgomery et al., 2013; Celius, 2017). Our results suggest that in addition to pre-existing infections, MS disease course or disability can serve as disease specific predictors to identify at-risk patients who could potentially benefit from mitigation strategies to prevent serious infections. Proposed mitigation strategies range from DMT targeted strategies focusing on dose/frequency adjustment, on-treatment prophylaxis and monitoring, to more general preventive measures including vaccination and behavioural modification strategies (Smith and Kister, 2021). We further demonstrate that the magnitude of associations are similar for measures of MS disease course and EDSS score. This is of potential clinical importance as assessment of disease course requires access to multiple sources of information and an extended period of observation (Lublin et al., 2014), while EDSS can be readily assessed in the clinic and used to identify patients at greater risk of serious infection. In light of the COVID-19 pandemic, a recent study (Louapre et al., 2020) identified higher neurological disability assessed by EDSS as an independent risk factor for COVID-19 complications, highlighting the importance of strengthening precautionary measures among patients with greater disability to limit the risk of infections. Our study extends these observations to serious infections in general, among patients treated with and without DMTs.

## 5 Conclusions

Compared to people without MS, the risk of serious infections is increased among those with a progressive disease course and higher EDSS score (which may be more readily available than disease course in some patients), independently of DMT use. The risk patterns observed highlight the need to further examine which patient management strategies including general preventive measures (including vaccination and simple hygiene measures) and DMT specific measures could be used to alleviate the excess risk of serious infections among MS patients with a progressive course or greater disability.

## Funding

This study was supported by grants from the UK 10.13039/501100000269Economic and Social Research Council (ESRC) to the International Centre for Life Course Studies (ES/R008930/1) and *Nyckelfonden.* The funders were not involved in the design and conduct of the study; collection, management, analysis, interpretation of the data; preparation, review, or approval of the manuscript; or decision to submit the manuscript for publication.

## Conflict of interest disclosures

SM has received research funding from Novartis, Roche and AstraZeneca, as well as serving on an advisory board for IQVIA and contributing to consensus meetings funded by Merck. FP has received research grants from Genzyme, Merck KGaA and Novartis, and fees for serving as Chair of DMC in clinical trials with Chugai, Lundbeck and Roche. TO has received honoraria for advisory boards and unrestricted MS research grants from Biogen, Novartis, Merck, Sanofi, and Roche. KAS receives research funding from the Multiple Sclerosis Society of Canada unrelated to this study. All other authors declare no conflict of interest.
