# Supplementary material for: Risk of serious infections in multiple sclerosis patients by disease course and disability status: Results from a Swedish register-based study
Source: Brain Behav Immun Health. 2022 May 11;22:100470. doi: 10.1016/j.bbih.2022.100470 (PMC9123212; doi:10.1016/j.bbih.2022.100470)
Supplement: Multimedia component 1 [file mmc1.docx]

**SUPPLEMENTARY FILE**

**Supplementary Figure 1.** Absolute rates of serious infection by attained age in MS patient groups and matched comparators of the general population.


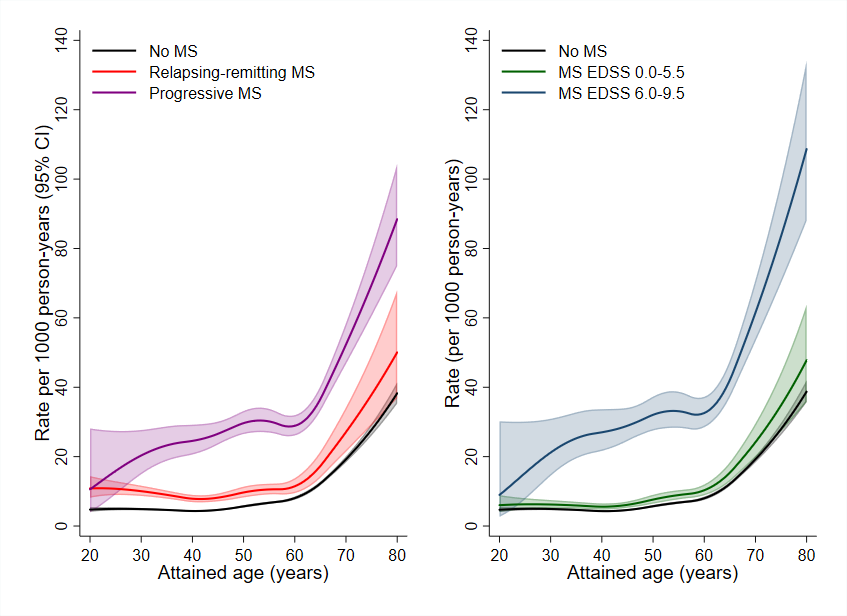


Absolute rates of serious infection by attained age in MS patient groups stratified by disease course and disability status and matched general population comparator individuals are derived from flexible parametric models. Abbreviations: MS = multiple sclerosis; EDSS = Expanded Disability Status Scale; CI = confidence interval.

**Supplementary Table 1.** Internal classification of diseases (ICD) codes for infections, overall and by site.

| **Any infection** | **ICD-version** | **Diagnostic code** |
| --- | --- | --- |
|  | ICD-9 | 001-134, 320, 380-383, 460-466, 480-487, 491B, 566, 567A, 567B, 590, 595, 680-686, 711A, 730, 770, 771 |
|  | ICD-10 | A00-B99, G00-G02, G04.2, G06-G07, H66, H70.0, H70.1, J00-J22, J32, J35.0, L00-L04, L08, L30.3, M00, M01, N10, N11, P23, P35-P39 |
| **Site of infection** | **ICD-version** | **Diagnostic code** |
| Respiratory infection | ICD-9 | 010-012, 031A, 033-034, 052A, 055B, 112E, 122B, 460-466, 480-487, 491B |
|  | ICD-10 | A15-A16, A20.2, A21.2, A22.1, A31.0, A37, A38, A48.1, B00.2,  B01.2, B05.2, B27, B37.1, B39-B42, B44, B45.0, B46.0, B58.3, B59, J00-J06, J10-J16, J18, J20-J22, J32, J35.0 |
| Sepsis | ICD-9 | 036A, 038, 112F |
|  | ICD-10 | A02.1, A20.7, A22.7, A24.1, A26.7, A28.2, A32.7, A39.2, A40, A41, A42.7, A48.3, B37.7 |
| Central nervous system infection | ICD-9 | 006F, 013, 036A-036B, 090E, 094X, 045-049, 052B, 053A, 053B, 054D, 055A, 056A, 062-064, 071, 072B-072C, 094, 320 |
|  | ICD-10 | A06.6, A17, A20.3, A22.8, A32.1, A39.0, A80-89, B00.3-B00.4, B01.0-B01.1, B02.0-B02.1, B05.0-B05.1, B06.0, B22.0, B26.1- B26.2, B37.5, B38.4, B43.1, B45.1, B46.1, B50.0, B57.4, B58.2, B60.2, B69.0, B83.2, G00-G02, G04.2, G06-G07 |
| Skin infection | ICD-9 | 017A, 031B, 035, 050-057, 074D, 091D, 110-111, 112D, 680-686 |
|  | ICD-10 | A18.4, A20.0, A22.0, A26.0, A31.1, A32.0, A36.3, A46, B00-B09, B35-36, B37.2, B43.0, B43.2, B45.2, B46.3, B55.1, L00-L04, L08 |
| Urinary tract infection | ICD-9 | 016A-016B, 590, 595 |
|  | ICD-10 | N10, N11, N390 |
| Gastrointestinal infection | ICD-9 | 001-009, 014, 123, 127, 129, 566, 567A, 567B |
|  | ICD-10 | A00-A09, A18.3, B68.0, B78.0, B82.9 |

**Supplementary Table 2.** Associations of MS disease course and disability with risk of any serious infection stratified by sex, overall and by attained age.

|  |  | Overall | | Attained age | | | | | |
| --- | --- | --- | --- | --- | --- | --- | --- | --- | --- |
|  |  |  | | < 40 yrs | | 40-60 yrs | | >60 yrs | |
|  | Total N | N events | HR (95% CI) | N events | HR (95% CI) | N events | HR (95% CI) | N events | HR (95% CI) |
| **Females** |  |  |  |  |  |  |  |  |  |
| No MS | 61120 | 3804 | REF | 990 | REF | 1615 | REF | 1199 | REF |
| MS clinical course |  |  |  |  |  |  |  |  |  |
| Relapsing-remitting | 4175 | 372 | 1.65 (1.48; 1.84) | 161 | 1.79 (1.51; 2.11) | 167 | 1.63 (1.39; 1.91) | 44 | 1.41 (1.05; 1.91) |
| Progressive | 1937 | 436 | 3.25 (2.94; 3.60) | 27 | 3.00 (2.04; 4.41) | 210 | 3.65 (3.15; 4.22) | 199 | 2.53 (2.18; 2.94) |
| MS EDSS score |  |  |  |  |  |  |  |  |  |
| 0.0-5.5 (mild/moderate) | 4292 | 273 | 1.12 (0.99; 1.27) | 87 | 1.02 (0.82; 1.27) | 128 | 1.18 (0.98; 1.41) | 58 | 1.17 (0.90; 1.53) |
| 6.0-9.5 (severe) | 1121 | 268 | 3.84 (3.38; 4.35) | 23 | 4.20 (2.77; 6.36) | 122 | 4.62 (3.84; 5.57) | 123 | 3.19 (2.65; 3.85) |
| **Males** |  |  |  |  |  |  |  |  |  |
| No MS | 25480 | 1772 | REF | 314 | REF | 798 | REF | 660 | REF |
| MS clinical course |  |  |  |  |  |  |  |  |  |
| Relapsing-remitting | 1489 | 164 | 2.10 (1.78; 2.46) | 73 | 2.86 (2.22; 3.69) | 71 | 1.76 (1.38; 2.25) | 20 | 1.95 (1.25; 3.06) |
| Progressive | 1059 | 365 | 4.81 (4.29; 5.40) | 37 | 8.09 (5.69;11.50) | 184 | 5.17 (4.40; 6.08) | 144 | 3.48 (2.90; 4.18) |
| MS EDSS score |  |  |  |  |  |  |  |  |  |
| 0.0-5.5 (mild/moderate) | 1588 | 167 | 1.76 (1.50; 2.07) | 49 | 2.01 (1.48; 2.71) | 78 | 1.60 (1.26; 2.02) | 40 | 1.91 (1.38; 2.63) |
| 6.0-9.5 (severe) | 625 | 181 | 5.24 (4.49; 6.12) | 21 | 8.20 (5.20;12.95) | 90 | 6.18 (4.96; 7.70) | 70 | 3.99 (3.11; 5.12) |

Hazard ratios comparing rates of serious infections in MS patient groups by disease course and disability with rates in matched individuals from the general population without MS (= reference), analyses stratified by sex. All hazard ratios are derived from flexible parametric models and are adjusted for matching factors (age, sex and region of residence) and educational attainment and calendar period of cohort entry.

Abbreviations: MS = multiple sclerosis; EDSS = Expanded Disability Status Scale; HR = hazard ratio; CI = confidence interval; REF = reference category.

**Supplementary Table 3.** Absolute rates of serious infections by disease course and disability status, overall and by attained age.

|  | Rate per 1000 person-years (95% CI) | | | |
| --- | --- | --- | --- | --- |
|  | Overall | Attained age |  |  |
|  |  | < 40 years | 40-60 years | >60 years |
| No MS | 6.75 (6.57; 6.92) | 4.77 (4.52; 5.04) | 5.65 (5.43; 5.88) | 14.70 (14.05; 15.39) |
| MS clinical course |  |  |  |  |
| Relapsing-remitting | 9.82 (9.02; 10.69) | 9.57 (8.42; 10.88) | 8.86 (7.80; 10.06) | 19.43 (15.21; 24.82) |
| Progressive | 32.64 (30.46; 34.98) | 21.72 (17.00; 27.76) | 25.33 (22.95; 27.96) | 41.71 (37.52; 46.37) |
| MS EDSS score |  |  |  |  |
| 0.0-5.5 (mild/moderate) | 7.68 (6.99; 8.43) | 5.91 (5.00; 7.00) | 7.13 (6.22; 8.17) | 18.13 (14.88; 22.10) |
| 6.0-9.5 (severe) | 36.44 (33.22; 39.97) | 25.17 (18.73; 33.82) | 31.01 (27.10; 35.47) | 51.66 (44.87; 59.49) |

Rates of serious infections by disease course and disability status and in matched comparators from the general population.

Abbreviations: MS = multiple sclerosis; EDSS = Expanded Disability Status Scale; CI = confidence interval.

**Supplementary Table 4.** Associations of MS disease course and disability with overall risk of any serious infection, stratified by type.

|  | Overall | |
| --- | --- | --- |
|  | Rate per 1000 person-years (95% CI) | HR (95% CI) |
| **Any serious infection** |  |  |
| No MS | 6.75 (6.57; 6.92) | REF |
| MS clinical course |  |  |
| Relapsing-remitting | 9.82 (9.02; 10.69) | 1.77 (1.62; 1.93) |
| Progressive | 32.64 (30.46; 34.98) | 3.80 (3.52; 4.09) |
| MS EDSS score |  |  |
| 0.0-5.5 (mild/moderate) | 7.68 (6.99; 8.43) | 1.30 (1.18; 1.43) |
| 6.0-9.5 (severe) | 36.44 (33.22; 39.97) | 4.26 (3.87; 4.70) |
| **Respiratory infection** |  |  |
| No MS | 3.13 (3.01; 3.25) | REF |
| MS clinical course |  |  |
| Relapsing-remitting | 3.68 (3.21; 4.22) | 1.48 (1.29; 1.71) |
| Progressive | 16.35 (14.89; 17.95) | 3.90 (3.51; 4.32) |
| MS EDSS score |  |  |
| 0.0-5.5 (mild/moderate) | 3.14 (2.72; 3.62) | 1.19 (1.02; 1.38) |
| 6.0-9.5 (severe) | 18.23 (16.15; 20.57) | 4.33 (3.80; 4.92) |
| **Sepsis** |  |  |
| No MS | 0.83 (0.77; 0.89) | REF |
| MS clinical course |  |  |
| Relapsing-remitting | 1.04 (0.80; 1.34) | 1.84 (1.41; 2.40) |
| Progressive | 5.93 (5.10; 6.91) | 4.67 (3.94; 5.55) |
| MS EDSS score |  |  |
| 0.0-5.5 (mild/moderate) | 0.94 (0.73; 1.22) | 1.43 (1.09; 1.87) |
| 6.0-9.5 (severe) | 6.64 (5.47; 8.07) | 5.34 (4.32; 6.59) |
| **CNS infection** |  |  |
| No MS | 0.19 (0.16; 0.22) | REF |
| MS clinical course |  |  |
| Relapsing-remitting | 0.24 (0.14; 0.41) | 1.35 (0.78; 2.33) |
| Progressive | 0.45 (0.26; 0.78) | 2.28 (1.28; 4.05) |
| MS EDSS score |  |  |
| 0.0-5.5 (mild/moderate) | 0.18 (0.10; 0.33) | 0.97 (0.52; 1.79) |
| 6.0-9.5 (severe) | 0.50 (0.25; 1.00) | 2.51 (1.23; 5.15) |
| **Skin infection** |  |  |
| No MS | 0.98 (0.91; 1.05) | REF |
| MS clinical course |  |  |
| Relapsing-remitting | 1.67 (1.37; 2.04) | 2.10 (1.70; 2.61) |
| Progressive | 4.61 (3.88; 5.48) | 3.58 (2.96; 4.32) |
| MS EDSS score |  |  |
| 0.0-5.5 (mild/moderate) | 1.23 (0.98; 1.55) | 1.43 (1.12; 1.81) |
| 6.0-9.5 (severe) | 4.73 (3.76; 5.95) | 3.67 (2.88; 4.67) |
| **Urinary tract infection** |  |  |
| No MS | 1.70 (1.61; 1.79) | REF |
| MS clinical course |  |  |
| Relapsing-remitting | 3.70 (3.23; 4.24) | 2.89 (2.50; 3.35) |
| Progressive | 22.34 (20.59; 24.24) | 9.34 (8.45;10.32) |
| MS EDSS score |  |  |
| 0.0-5.5 (mild/moderate) | 3.28 (2.85; 3.77) | 2.36 (2.03; 2.74) |
| 6.0-9.5 (severe) | 24.64 (22.09; 27.49) | 9.92 (8.77;11.23) |

**Supplementary Table 4.** continued.

|  | Overall | |
| --- | --- | --- |
|  | Rate per 1000 person-years (95% CI) | HR (95% CI) |
| **Gastrointestinal infection** |  |  |
| No MS | 0.91 (0.84; 0.97) | REF |
| MS clinical course |  |  |
| Relapsing-remitting | 1.27 (1.00; 1.59) | 1.58 (1.24; 2.02) |
| Progressive | 4.07 (3.39; 4.89) | 3.70 (3.03; 4.53) |
| MS EDSS score |  |  |
| 0.0-5.5 (mild/moderate) | 0.93 (0.72; 1.21) | 1.12 (0.85; 1.47) |
| 6.0-9.5 (severe) | 4.65 (3.69; 5.86) | 4.10 (3.20; 5.24) |

Rates and hazard ratios comparing overall risk of site-specific serious infections in MS patient groups by disease course and disability using matched individuals from the general population without MS as a reference. All rates and hazard ratios are derived from flexible parametric models with attained age as underlying time scale. Hazard ratios are adjusted for the matching factors (age, sex and region of residence) and educational attainment and calendar period of cohort entry.

Abbreviations: MS = multiple sclerosis; EDSS = Expanded Disability Status Scale; HR = hazard ratio; CI = confidence interval; REF = reference category.

**Supplementary Table 5.** Associations of MS disease course and disability status with infection-related mortality and hospital admission, overall and by attained age.

|  |  | Overall | | Attained age | | | | | |
| --- | --- | --- | --- | --- | --- | --- | --- | --- | --- |
|  |  |  |  | < 40 yrs | | 40-60 yrs | | >60 yrs | |
|  | Total N | N events | HR (95% CI) | N events | HR (95% CI) | N events | HR (95% CI) | N events | HR (95% CI) |
| Infection-related mortality |  |  |  |  |  |  |  |  |  |
| No MS | 86600 | 364 | REF | 13 | REF | 121 | REF | 230 | REF |
| MS clinical course |  |  |  |  |  |  |  |  |  |
| Relapsing-remitting | 5664 | 7 | 0.58 (0.28; 1.24) | 0 | NA | 4 | 0.65 (0.24; 1.77) | 3 | 0.71 (0.23; 2.24) |
| Progressive | 2996 | 86 | 3.81 (3.00; 4.83) | 4 | 27.04 (8.00; 91.45) | 30 | 4.86 (3.24; 7.29) | 52 | 2.94 (2.17; 3.98) |
| MS EDSS score |  |  |  |  |  |  |  |  |  |
| 0.0-5.5 (mild/moderate) | 5677 | 8 | 0.49 (0.24; 0.98) | 0 | NA | 5 | 0.66 (0.27; 1.62) | 3 | 0.39 (0.12; 1.22) |
| 6.0-9.5 (severe) | 1912 | 64 | 5.01 (3.83; 6.56) | 2 | 14.58 (3.03;70.15) | 23 | 7.49 (4.77;11.77) | 39 | 4.00 (2.84; 5.64) |
| Infection-related hospital admission |  |  |  |  |  |  |  |  |  |
| No MS | 86600 | 5492 | REF | 1298 | REF | 2381 | REF | 1813 | REF |
| MS clinical course |  |  |  |  |  |  |  |  |  |
| Relapsing-remitting | 5664 | 534 | 1.78 (1.63; 1.95) | 234 | 2.04 (1.77; 2.34) | 237 | 1.68 (1.47; 1.92) | 63 | 1.57 (1.22; 2.02) |
| Progressive | 2996 | 797 | 3.85 (3.57; 4.15) | 64 | 4.70 (3.64; 6.06) | 392 | 4.26 (3.82; 4.75) | 341 | 2.90 (2.58; 3.26) |
| MS EDSS score |  |  |  |  |  |  |  |  |  |
| 0.0-5.5 (mild/moderate) | 5677 | 438 | 1.31 (1.19; 1.44) | 136 | 1.25 (1.04; 1.49) | 204 | 1.31 (1.13; 1.51) | 98 | 1.43 (1.16; 1.75) |
| 6.0-9.5 (severe) | 1912 | 448 | 4.34 (3.93; 4.78) | 44 | 5.45 (4.02; 7.39) | 212 | 5.25 (4.55; 6.04) | 192 | 3.49 (3.00; 4.05) |

Hazard ratios comparing rates of infection-related mortality and hospital admission in MS patient groups by disease course and disability with rates in matched individuals from the general population without MS (= reference). All hazard ratios are derived from flexible parametric models and are adjusted for matching factors (age, sex and region of residence) and educational attainment and calendar period of cohort entry.

Abbreviations: MS = multiple sclerosis; EDSS = Expanded Disability Status Scale; HR = hazard ratio; CI = confidence interval; REF = reference category.

**Supplementary Table 6.** Associations of MS disease course and disability with risk of any serious infection, overall and by attained age – sensitivity analyses including main diagnoses only.

|  |  | Overall | | Attained age | | | | | |
| --- | --- | --- | --- | --- | --- | --- | --- | --- | --- |
|  |  |  |  | < 40 yrs | | 40-60 yrs | | >60 yrs | |
|  | Total N | N events | HR (95% CI) | N events | HR (95% CI) | N events | HR (95% CI) | N events | HR (95% CI) |
| No MS | 86600 | 4080 | REF | 995 | REF | 1772 | REF | 1313 | REF |
| MS clinical course |  |  |  |  |  |  |  |  |  |
| Relapsing-remitting | 5664 | 419 | 1.84 (1.67; 2.04) | 181 | 2.05 (1.75; 2.40) | 198 | 1.89 (1.63; 2.18) | 40 | 1.33 (0.97; 1.82) |
| Progressive | 2996 | 653 | 4.19 (3.85; 4.56) | 55 | 5.09 (3.86; 6.70) | 330 | 4.77 (4.23; 5.37) | 268 | 3.04 (2.66; 3.47) |
| MS EDSS score |  |  |  |  |  |  |  |  |  |
| 0.0-5.5 (mild/moderate) | 5677 | 329 | 1.30 (1.16; 1.46) | 99 | 1.18 (0.96; 1.45) | 162 | 1.39 (1.18; 1.63) | 68 | 1.33 (1.04; 1.70) |
| 6.0-9.5 (severe) | 1912 | 379 | 4.74 (4.26; 5.28) | 38 | 5.97 (4.30; 8.29) | 188 | 6.06 (5.20; 7.05) | 153 | 3.57 (3.02; 4.23) |

Hazard ratios comparing rates of serious infection (including primary diagnoses only) in MS patient groups by disease course and disability with rates in matched individuals from the general population without MS (= reference). All hazard ratios are derived from flexible parametric models and are adjusted for matching factors (age, sex and region of residence) and educational attainment and calendar period of cohort entry.

Abbreviations: MS = multiple sclerosis; EDSS = Expanded Disability Status Scale; HR = hazard ratio; CI = confidence interval; REF = reference category.

**Supplementary Table 7.** Associations of MS disease course and disability with risk of serious infection stratified by disease modifying therapy, overall and by attained age – sensitivity analysis including main diagnoses only.

|  |  | Overall | | Attained age | | | | | |
| --- | --- | --- | --- | --- | --- | --- | --- | --- | --- |
|  |  |  |  | < 40 yrs | | 40-60 yrs | | >60 yrs | |
|  | Total N | N events | HR (95% CI) | N events | HR (95% CI) | N events | HR (95% CI) | N events | HR (95% CI) |
| Never DMT |  |  |  |  |  |  |  |  |  |
| MS clinical course |  |  |  |  |  |  |  |  |  |
| Relapsing-remitting | 2601 | 174 | 1.51 (0.98; 1.77) | 45 | 1.32 (0.98; 1.77) | 102 | 1.67 (1.36; 2.03) | 27 | 1.45 (0.99; 2.12) |
| Progressive | 1667 | 382 | 4.24 (4.78; 9.55) | 35 | 6.75 (4.78; 9.55) | 199 | 4.61 (3.97; 5.35) | 148 | 3.06 (2.58; 3.63) |
| MS EDSS score |  |  |  |  |  |  |  |  |  |
| 0.0-5.5 (mild/moderate) | 2706 | 144 | 1.08 (0.92; 1.28) | 20 | 0.62 (0.40; 0.97) | 83 | 1.20 (0.97; 1.50) | 41 | 1.28 (0.94; 1.76) |
| 6.0-9.5 (severe) | 954 | 242 | 4.86 (4.26; 5.54) | 25 | 8.42 (5.62;12.60) | 116 | 5.58 (4.62; 6.75) | 101 | 3.92 (3.19; 4.80) |
|  |  |  |  |  |  |  |  |  |  |
| Ever DMT |  |  |  |  |  |  |  |  |  |
| MS clinical course |  |  |  |  |  |  |  |  |  |
| Relapsing-remitting | 2592 | 203 | 2.39 (2.07; 2.76) | 127 | 2.53 (2.10; 3.05) | 73 | 2.29 (1.81; 2.90) | 3 | 1.49 (0.48; 4.65) |
| Progressive | 522 | 69 | 4.76 (3.74; 6.04) | 16 | 3.92 (2.39; 6.43) | 46 | 5.22 (3.89; 7.01) | 7 | 3.63 (1.72; 7.65) |
| MS EDSS score |  |  |  |  |  |  |  |  |  |
| 0.0-5.5 (mild/moderate) | 2396 | 142 | 1.69 (1.43; 2.00) | 77 | 1.60 (1.26; 2.01) | 60 | 1.81 (1.40; 2.35) | 5 | 2.18 (0.90; 5.27) |
| 6.0-9.5 (severe) | 434 | 55 | 5.84 (4.47; 7.63) | 12 | 4.00 (2.26; 7.08) | 39 | 7.65 (5.56;10.52) | 4 | 3.10 (1.16; 8.29) |

Hazard ratios comparing rates of serious infection (including primary diagnoses only) in MS patient groups by disease course and disability and stratified by disease modifying therapy with rates in matched individuals from the general population without MS (= reference). All hazard ratios are derived from flexible parametric models and are adjusted for matching factors (age, sex and region of residence) and educational attainment and calendar period of cohort entry.

Abbreviations: MS = multiple sclerosis; EDSS = Expanded Disability Status Scale; HR = hazard ratio; CI = confidence interval; REF = reference category, DMT, disease modifying therapy. DMT is defined as Rituximab, Natalizumab, Alemtuzumab, Dimethyl Fumarate or Fingolimod.

**Supplementary Table 8.** Associations of MS disease course and disability with risk of infection-related hospital admission, overall and by attained age – sensitivity analysis requiring at least 2 diagnoses 6 months apart.

|  |  | Overall | | Attained age | | | | | |
| --- | --- | --- | --- | --- | --- | --- | --- | --- | --- |
|  |  |  | | < 40 yrs | | 40-60 yrs | | >60 yrs | |
|  | Total N | N events | HR (95% CI) | N events | HR (95% CI) | N events | HR (95% CI) | N events | HR (95% CI) |
| No MS | 86600 | 1301 | REF | 199 | REF | 555 | REF | 547 | REF |
| MS clinical course |  |  |  |  |  |  |  |  |  |
| Relapsing-remitting | 5664 | 106 | 1.68 (1.38; 2.05) | 52 | 3.10 (2.28; 4.21) | 41 | 1.31 (0.96; 1.81) | 13 | 1.12 (0.65; 1.95) |
| Progressive | 2996 | 380 | 6.42 (5.71; 7.22) | 41 | 14.47 (10.10; 20.74) | 193 | 7.24 (6.11; 8.59) | 146 | 3.57 (2.97; 4.29) |
| MS EDSS score |  |  |  |  |  |  |  |  |  |
| 0.0-5.5 (mild/moderate) | 5880 | 89 | 1.21 (0.97; 1.50) | 28 | 1.74 (1.17; 2.59) | 36 | 1.01 (0.72; 1.41) | 25 | 1.21 (0.81; 1.81) |
| 6.0-9.5 (severe) | 1746 | 206 | 6.66 (5.73; 7.73) | 24 | 15.33 (9.92; 23.69) | 100 | 9.36 (7.54; 11.61) | 82 | 4.23 (3.35; 5.34) |

Hazard ratios comparing rates of infection-related hospital admission in MS patient groups by disease course and disability status with rates in matched individuals from the general population without MS (= reference). All hazard ratios are derived from flexible parametric models and are adjusted for matching factors (age, sex and region of residence) and educational attainment and calendar period of cohort entry.

Abbreviations: MS = multiple sclerosis; EDSS = Expanded Disability Status Scale; HR = hazard ratio; CI = confidence interval; REF = reference category.
